# Longitudinal associations between screen time activities and working memory neural signatures in adolescents: Sex differences and contextual moderation

**DOI:** 10.1016/j.dcn.2026.101836

**Published:** 2026-10-06

**Authors:** Sahithi Arekatla, Anthony Barrows, Leigh-Anne Cioffredi, Matthew D. Albaugh, Nicholas Allgaier, Mckay Allred, David Baranger, Alexandra Potter, Hugh P. Garavan, Bader Chaarani

**Affiliations:** a University of Vermont, Department of Psychiatry, Burlington, Vermont, United States; b Medical College of Wisconsin, Department of Pharmacology and Toxicology, Milwaukee, WI, United States

**Keywords:** Screen Media Activity, Working Memory, Task fMRI, Neural Signatures, Mental Health, Family conflict

## Abstract

**Introduction:**

Adolescent screen media activity (SMA) has increased, yet its longitudinal associations with neurocognitive development and mental health remain unclear across activities and contexts.

**Methods:**

This longitudinal cohort study used data from the Adolescent Brain Cognitive Development (ABCD) Study (release 6.0) with four assessments (baseline, year 2, year 4, and year 6). Participants were recruited across 21 U.S. sites to reflect demographic diversity. Children with valid neuroimaging and behavioral data were included, adjusting for demographic, behavioral, and psychiatric confounders. Working memory (WM) neural signature (NS) scores were derived using elastic net classification of EN-back fMRI activation during 0-back and 2-back conditions. SMA included time spent video gaming, watching television, texting, and social media. Cognitive performance was measured using EN-back accuracy, and mental health symptoms using Child Behavior Checklist internalizing and externalizing scores. Moderation analyses examined family conflict, sleep disturbance, and physical activity.

**Results:**

The analytic sample included 9931 participants (47.2% female). Higher NS scores were associated with better 0-back and 2-back accuracy. Greater video gaming, texting, and television watching were associated with lower NS scores. Sex-stratified analyses showed among males, more screen time was associated with lower NS scores, a multivariate measure of how separable neural activation patterns are between the 2-back and 0-back conditions. These associations were specific to the EN-back task and were adjusted for general cognitive ability. Higher degrees of family conflict increased the association between NS with texting, and video gaming and externalizing symptoms among males. Sleep disturbance and physical activity also moderated associations with internalizing and externalizing symptoms, with sex-specific patterns.

**Conclusions:**

WM-related neural function was associated with cognitive performance and SMA engagement, differing by sex and environmental context, highlighting complex associations between neurocognitive development, media use, and mental health.

## 1 Introduction

Adolescents today are immersed in screen-based technologies, including smartphones, streaming platforms, video games, and social media. National trends show increased adolescent screen use over the past decade (Average Screen Time Statistics, 2026, Massarat EAV Risa Gelles Watnick and Navid. Teens, Social Media and Technology, 2022), with screen engagement occupying a growing proportion of adolescents’ daily routines. These trends have raised concerns about adverse cognition, mental health (Zablotsky et al., 2025), and behavioral outcomes. Consistent with these trends, recent national surveys indicate that over half (50.4%) of U.S. adolescents reported engaging in four or more hours of daily recreational screen use between 2021 and 2023 (Zablotsky et al., 2024). This widespread screen exposure underscores the need to understand its developmental consequences in youth. Evidence suggests that screen-related outcomes vary by activity type, engagement level, and individual differences. For instance high Screen Media Activity (SMA) in general has been linked to less efficient cognitive control (Marciano et al., 2021), whereas video gaming has been associated with faster reaction times, better WM (Chaarani et al., 2022), enhanced visuospatial processing (Chaarani et al., 2022, Green and Bavelier, 2006), greater frontoparietal engagement during visuospatial tasks (Richlan et al., 2018), and increased WM-area activation during the EN-back task (Chaarani et al., 2022). In contrast, social media use has been shown to acutely impair WM (Mittal et al., 2026) and a 2024 study found active screen time related to better visual WM (Sarvajna et al., 2024).

Behavioral studies associate high screen use, especially passive media consumption like social media and television watching with increased internalizing and externalizing symptoms (Eirich et al., 2022), sleep disturbances (Parent et al., 2016) and depressive symptoms during adolescence (Boers et al., 2019). Similarly, an experimental study found that social media use was linked to higher depression and anxiety scores, with WM performance mediating the relationship between social media usage and academic outcomes (Almarzouki et al., 2022). However, interactive media such as gaming for prolonged hours has been linked to higher depression and ADHD symptoms. This provides a pathway by which screen activities that place high or low demands on WM-related neural systems could extend beyond cognitive performance to shape emotional and behavioral functioning, motivating our use of a WM neural signature to link screen activity with mental health outcomes instead of treating cognitive findings and mental-health findings as two separate literatures.

With regard to SMA differences related to sex, surveys indicate higher total use in males whole spend over an hour more on screens than females (Rideout, 2019). Also, there is a qualitative developmental difference in terms of the SMA type, where for instance, video gaming rises sharply among males in preadolescence, whereas females tend to engage more in social media use and the gap widens further across early adolescence (Eirich et al., 2022). Consistently, boys show more signs of video game addiction, while girls show more signs of social media addiction (Bagot et al., 2022). Given this difference in sex-specific exposure to different screen activities and prior longitudinal work reporting that television, computer, and gaming use predicted better WM in young adult males but not females¹ ³, we tested sex-stratified models rather than treating sex purely as a covariate.

Although WM paradigms have been used in neuroimaging research on SMA use, no large-scale investigation has developed a neural signature (NS) of WM activation and examined how it relates to real-world patterns of screen use. NS is a classifier distinguishing high and low WM load during the emotional EN-back task, capturing how separable an individual’s neural responses are across task conditions and are more reliable and strongly related to behavior, cognition, and psychopathology than traditional region-based activation estimates (Baranger et al., 31, 2025), allowing for the investigation of associations between screen activity and WM neural systems.

There remains an opportunity to better understand the relationship between screen activity, its association with cognitive constructs and the interactive association with behavioral outcomes, particularly given emerging evidence that environmental factors may moderate these effects, such as sleep (van Tetering et al., 2025), family conflict (Yau et al., 2021, Liu et al., 2019) and physical activity (Yang and Sun, 2025). In fact, screen use can disrupt sleep through delayed circadian phase shifts and pre-sleep arousal, whereas disturbed sleep has been linked to poorer cognitive functioning, potentially through altered frontoparietal activation during WM tasks, particularly among girls (Yan et al., 2024). Recent work in the ABCD sample further suggests that neural reward sensitivity shapes the prospective association between social media use and sleep duration (Kiss et al., 2026), indicating that individual differences in brain function may influence the impact of screen-related behaviors on sleep. Likewise, greater screen time has been associated with shorter sleep duration and poorer attention and WM, with these relationships varying by digital content type and age (Toth et al., 2025). Together, these findings suggest that sleep may function not only as a correlate of screen use but also as a contextual factor that influences the strength of associations between screen-related behaviors and neural mechanisms supporting WM Accordingly, we examined sleep disturbance as a moderator of the association between screen time and WM neural signatures rather than as a mediating pathway.

Here, using a large data set of 9- and 10-year-old children from the Adolescent Brain Cognitive Development (ABCD) study, (Casey et al., 2018) to create a multivariate WM NS score. Subsequently, we examined associations of these individual NS scores with WM performance, self-reported screen time activities (video games, television, social media, texting), and key confounding factors (such as sleep disturbance, family conflict and physical activity). We further tested sex differences and examined whether sleep disturbance, family conflict, and physical activity moderated the relationships between NS, screen use, and internalizing and externalizing symptomatology. We hypothesized that: 1) task-performance will be associated with better higher NS score 2) different screen activities will have divergent associations with the WM neural signature based on their inherent cognitive demands (e.g., interactive versus passive media); (3) variations in this WM neural signature will in turn correlate with internalizing and externalizing symptoms, acting as a neurocognitive bridge between specific screen behaviors and mental health outcomes; and (4) these neurocognitive and behavioral pathways will differ significantly by sex. We also anticipate, based on the established evidence previously mentioned, that the strength of these associations will be moderated by contextual environmental and lifestyle factors, specifically family conflict, sleep disturbances, and physical activity.

## 2 Methods

### 2.1 Sample

Participants were enrolled in the ongoing longitudinal ABCD Study. The ABCD sample was largely recruited through public, private, and charter elementary schools. The ABCD study adopted a population neuroscience approach to recruitment (Falk et al., 2013, Paus, 2013) by using epidemiologically informed procedures to ensure demographic variation in its sample that would mirror the variation in the US population of 9- and 10-year-olds (Garavan et al., 2018). A probability sampling of schools was conducted within the defined catchment areas of the study’s nationally distributed set of 21 recruitment sites in the US. All 9–10 year old children in each sampled school were invited to participate after classroom-based presentations, distribution of study materials, and telephone screening for eligibility. Exclusions included common MRI contraindications (such as cardiac pacemakers and defibrillators, internal pacing wires, cochlear and metallic implants, and Swan-Ganz catheters), inability to understand or speak English fluently, uncorrected vision, hearing or sensorimotor impairments, history of major neurologic disorders, gestational age less than 28 weeks, birth weight less than 1200 g, birth complications that resulted in hospitalization for more than 1 month, current diagnosis of schizophrenia, moderate or severe autism spectrum disorder, history of traumatic brain injury, or unwillingness to complete assessments.

The study data were from the 6.0 data release. The ABCD study’s de-identified data, including all assessment domains, are released annually to the research community. Information on how to access ABCD study data through the NIH Brain Development Cohorts (NBDC) data sharing platform is available on https://www.nbdc-datahub.org/. The ABCD Study recruited 11,874 healthy children, ages 9–10, to be followed into early adulthood. The ABCD study was approved by the appropriate institutional review boards: most ABCD research sites rely on a central Institutional Review Board at the University of California, San Diego for the ethical review and approval of the research protocol, with a few sites obtaining local IRB approval. Participants provided written assent, and their legal guardians written consent, for participation. The child’s age and sex were reported by the parent at the baseline assessment.

### 2.2 Youth mental health problems

Mental health symptoms were assessed using the Child Behavior Checklist (CBCL) (Achenbach and Ruffle, 2000), a parent-report measure consisting of 113 items rated on a 3-point scale ranging from 0 (Not True) to 2 (Very True or Often True). In the ABCD Study, these data were obtained using a computerized version of the measure. Specifically, the CBCL yields dimensional assessments of common emotional and behavioral problems in children and adolescents. In the present study, we used raw scores from the broadband Internalizing and Externalizing scales.

### 2.3 Family environment, sleep problems and physical activity

Confounders included family conflict, sleep duration, and physical activity. Family conflict scores were derived from the conflict subscale of the Family Environment Scale (Moos and Moos, 1976), which was selected to assess a core aspect of social interaction and family climate (Zucker et al., 2018). The 9-item subscale assesses the extent of anger, criticism, fighting, competitiveness, yelling, and loss of temper within the family (e.g., “We fight a lot in our family”). Adolescents and parents indicated whether each item was true (1) or false (0). Prorated subscale scores were used to minimize missing data and were calculated by multiplying the raw summed score (after reverse coding where appropriate) by the total number of items (9) and dividing by the number of items completed. Participants who completed fewer than five items were coded as missing. The resulting scores ranged from 0 to 9, with higher values indicating greater family conflict, and were analyzed as a continuous variable.

Sleep characteristics were assessed using the parent-reported Sleep Disturbance Scale for Children (SDSC) (Bruni et al., 1996), a 27-item questionnaire rated on a 5-point Likert scale designed to characterize sleep problems in children. Total sleep time was derived from the item, “How many hours of sleep does your child get on most nights?”, with response options coded on a 5-point ordinal scale: 1 = 9–11 h, 2 = 8–9 h, 3 = 7–8 h, 4 = 5–7 h, and 5 = less than 5 h, with higher scores reflecting shorter sleep duration.

Physical activity was assessed using three items adapted from the Youth Risk Behavior Survey (YRBS) (Barch et al., 2018), (Eaton et al., 2012) where youth reported the number of days in the past week (range: 0–7 days) that they engaged in at least 60 min of physical activity per day and the number of days (range: 0–7 days) that they performed muscle-strengthening or toning exercises. These items were treated as continuous indicators of weekly physical activity.

### 2.4 fMRI acquisition and processing

High spatial and temporal resolution simultaneous multi-slice/multiband EPI task-based fMRI scans, with fast integrated distortion correction, were acquired to examine functional activity. For Siemens and GE 3 tesla scanners, the scanning parameters were: matrix of 90 × 90, 60 slices, FOV= 216 × 216, TE/TR (msec) = 800/30, flip angle= 52 °, and resolution (mm) = 2.4 × 2.4 × 2.4. The fMRI acquisitions (2.4 mm isotropic, TR = 800 ms) used multiband EPI with slice acceleration factor 6. The ABCD Data Analysis, Informatics, & Resource Center (DAIRC) performed centralized initial quality control and processing of the fMRI data. All MRI assessments were reviewed by a neuroradiologist for incidental findings. Using a combination of automated and manual methods, the fMRI datasets were quality controlled for problems such as acquisition protocol compliance, imaging artifacts, motion or file corruption. The full details of the imaging acquisition protocol were previously described in (Casey et al., 2018, Hagler et al., 2019).

### 2.5 fMRI EN-back task

The EN-back task was selected from the ABCD imaging battery to probe WM. Participants practiced the task prior to scanning to ensure that they understood the instructions and were familiar with the response collection device. The ABCD imaging protocol was designed to extend the benefits of high temporal and spatial resolution of imaging protocols of the Human Connectome Project (Hagler et al., 2019) with the multiple scanner systems of participating sites (Chaarani et al., 2021). In this WM block-design task, participants saw a series of stimuli and indicated whether each one was the same or different from the stimulus N items earlier (i.e., “N back”). The task included two conditions: a 2-back as the active condition and a 0-back as the baseline condition, which included similar visuo-motor demands but lower WM load. In the 0-back condition, participants indicated if each stimulus matched a single target presented at the beginning of the trial, thereby obviating the need to maintain and update a two-item WM load throughout the task. Responses on the 2-back and 0-back were input on a two-button keypad, with one button indicating the stimulus was a match and the other indicating no match. The EN-back consisted of two runs, each containing eight blocks of trials and four 15 s rest periods containing just a fixation cross. Blocks contained 10 trials lasting 2.5 s each and were preceded by a 2.5 s instruction screen indicating the condition for the upcoming block. Of the 10 trials in each block, 2 were targets, 2–3 were non-target lures, and the remainder were non-lures (i.e., stimuli only presented once). There were 160 trials in total with 96 unique stimuli of 4 different stimulus types (24 unique stimuli per type). Three-quarters of the stimulus types were human faces, demonstrating happy, fearful, or neutral facial expressions, with facial expression stimulus type held constant within each block. Contrasts of interest include 0-back vs. fixation, 2-back vs. fixation. The data used in this study were from four timepoints (baseline, year2, year4 and year6).

To assess behavioral task performance on the task, accuracy rates were computed for both the 2-back and 0-back conditions by calculating each participant’s hit rate, the proportion of targets for which the participant correctly indicated a match, and false alarm rate, the proportion of non-targets for which the participant incorrectly indicated a match or did not respond. Children were excluded from the analyses if accuracy was 0 or lower, consistent with our previous work (Chaarani et al., 2021).

### 2.6 Screen Time Survey

Participants were administered a screen time survey that asked how much time they spend engaged in different types of screen time on a typical weekday and a typical weekend day. The different screen time categories included: “Watch TV shows or movies?”; “Play video games on a computer, console, phone, or other device (Xbox, PlayStation, iPad)?”; “Text on a cell phone, tablet, or computer (eg, GChat, Whatsapp, etc.)?”; and “Visit social networking sites like Facebook, Twitter, Instagram, etc?”; For each of these activities, the participants responded with how much time they spent per weekday and weekend day doing them. They could answer none, less than 30 min, 30 min, 1 h, 2 h, 3 h, or more hours. For each participant, a total weekly screen time type’s score was calculated as ((weekday hours + minutes/60) × 5) + ((weekend day hours + minutes/60) × 2).

The other screen time variables included “Watching Videos” (only available from baseline to year2) later merged into Watching TV, “editing photos or videos on social media” only available for yr2, “Searching or browsing the internet” only available for year 2 and 3 and “video chatting” is highly skewed in initial years and its out of the scope for our current analyses.

### 2.7 Neural signature

A “neural signature”(NS) of WM was computed in-house from estimates of ABCD Study EN-Back task-related activation obtained from the curated release 6.0 (https://www.nbdc-datahub.org/abcd-release-6–0) similarly to methods described in Baranger et. al (Baranger et al., 31, 2025). Activation estimates were averaged across image acquisition runs for 2-back and 0-back conditions, parcellated into cortical (Destrieux et al., 2010) and subcortical (Fischl et al., 2002) regions of interest (ROIs). These estimates were residualized for association with participants’ age, sex, average framewise displacement during each scan and scanner serial number before model training.

Elastic Net logistic regression was used to obtain a multivariate signature of task activation by classifying each participant’s task-related activation map according to its task condition (i.e., 2-back or 0-back) using a nested cross-validation scheme. Regression beta weights were averaged across validation folds and subsequently Haufe-transformed (Haufe et al., 2014) to ensure that feature importance reflected underlying brain activation patterns rather than suppression effects due to correlated predictors. Estimates of fMRI task-related activation for the ABCD EN-Back task were pre-processed and labeled according to the steps described in (Hagler et al., 2019). Region of interest Beta weights were averaged across imaging runs. The whole brain activation maps 2 back minus 0back for baseline, year2, year4, year6 follow up are presented in Fig. 1. A total of 166 (Desikan et al., 2006) and ASEG subcortical (Fischl et al., 2002) were used as predictors to train a classifier using Elastic Net logistic regression. The data were first divided into 80% training and 20% testing partitions. Using the training partition, an Elastic Net logistic regression was fitted using an “inner” 3-fold cross validated random hyperparameter search implemented in Bennet et al. (2021) through the Python library Brain Predictability toolbox (Hahn et al., 2025). L1 ratios between 0.01 and 1 and C (inverse of regularization strength) between Log(10e-5) and Log(100,000) were randomly permuted for 100 iterations. This was repeated 5 times (i.e., 5-fold “outer” validation loop). The optimal model was the one from the 5 outer validation folds which maximized receiver operator characteristic area under the curve (ROC AUC).

**Fig. 1:**
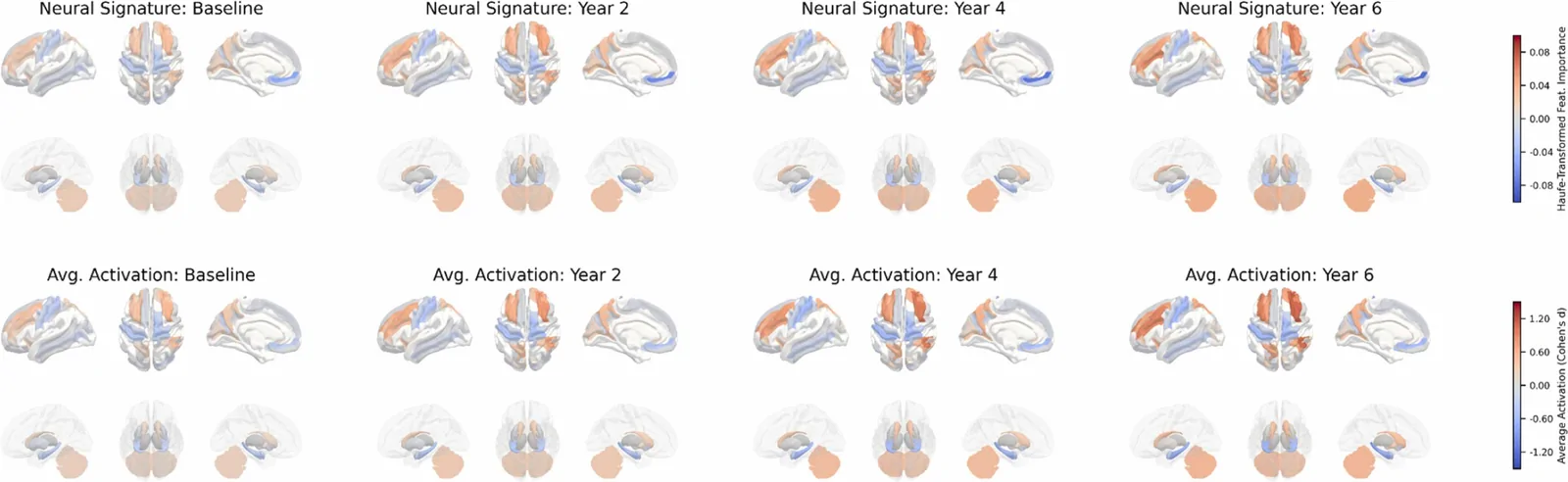
Longitudinal whole-brain maps of EN-back task 2-back minus 0-back contrast activation at baseline, year2, year4, year6 follow-up.

In order to obtain an axis of individual differences related to this NS, the trained classifier was used to make predictions from each participant’s activation maps. As the classifier was trained to differentiate the high-load (2-back) condition from the low-load (0-back), the resulting prediction probability reflects the extent to which a given map is likely to be from the high-load condition (i.e., prediction sensitivity). To introduce a component of specificity to these values, a prediction obtained using a given participant’s 0-back map was subtracted from the prediction obtained using that participant’s 2-back map. This difference in prediction for each participant was taken as a single, continuous axis of individual differences in EN-back-related brain activation.

### 2.8 Participant and imaging inclusion criteria

Participants were included if they had complete information on screen time activities (texting, social media, watching TV, and video games) for both weekdays and weekends at baseline, year2, and year4, and for weekends only at year6. Participants were excluded if their reported screen time exceeded an average of 75 h per week for any activity. For the EN-back analyses, participants were excluded if they failed the imaging quality control, which requires having 1) two fMRI runs per task, 2) hemispheric mean beta-weights within three standard deviations of the sample mean for each task, 4) at least 200 ° of freedom over the two scan runs, 5) mean framewise displacement < 0.9 mm for both runs. These QC steps were applied prior to generating NS scores.

### 2.9 Statistical analyses

Outcomes of interest included self-reported screen time activities (texting, social media, TV watching, and video games) and task accuracies of 0-back and 2-back conditions from the EN-back fMRI task. The primary independent variable was the NS score. All outcome and predictor variables were standardized (z-scored) prior to analysis.

Associations were tested using linear mixed-effects (LME) models, with random effects nested by participant and study site. To control for additional potential confounds, a set of covariates including age (linear and quadratic terms) to capture developmental change across timepoints, sex, race/ethnicity, head motion (as measured by mean framewise displacement), NIH-TB total composite score (IQ), sleep disturbance, physical activity, and family conflict were included in the models. FDR-corrected *p* values < .05 were considered statistically significant. All analyses were conducted in R using the lme4 package.

To further evaluate potential sex differences, sex-stratified analysis was performed. Separate models were run for males and females, each including the same set of covariates (excluding sex).

Interaction analyses were conducted using CBCL externalizing and internalizing scores as outcome variables which have been previously shown to be associated with the NS in this sample. Two-way interaction models tested associations between NS scores and each SMA (texting, social media, TV watching, and video games) including the same set of covariates mentioned above. Subsequently, three sets of three-way interaction models were run to assess whether these associations were moderated by a) physical activity, b) sleep disturbance, or c) family conflict, including all the covariates excluding the moderator. All models were further stratified by sex to test sex differences using the same set of covariates.

## 3 Results

### 3.1 Demographics

The final sample included a total of 9931 participants who completed the screen time survey and who had EN-back accuracy measured. Raw means for the demographics and confounding factors across screen time users and EN-back accuracy measures are detailed in Table 1.

**Table 1:** Raw means of the demographics and confounder variables in the sample (N = 9931). Higher sleep hours, family conflict and physical activity scores reflect less sleep hours, more family conflict and more physical activity.

Sample	Age (months)				Family Conflict	Physical Activity	Sleep hours	IQ	Sex (% Female)	Race (%by category)
	Baseline	Year 2	Year 4	Year 6						
Sample size N = 9931	120.06 (7.54)	144.20 (7.82)	170.01 (8.69)	192.97 (7.49)	1.98 (1.94)	3.72 (2.17)	1.97 (0.89)	104.78 (17.41)	47.20%	White: 57.1%, Black: 11.5%, Hispanic: 19.2%, Asian: 2%, Other: 10.2%

Time spent on media type varied substantially between sexes (Fig. 2). Specifically, more than 60% of males reported 0 h spent on social media and nearly half of males reported not texting. Conversely, female participants reported 0 h or less than 30 min much more frequently than males. This distinct exposure difference led us to complete sex stratified analyses for all objectives.

**Fig. 2:**
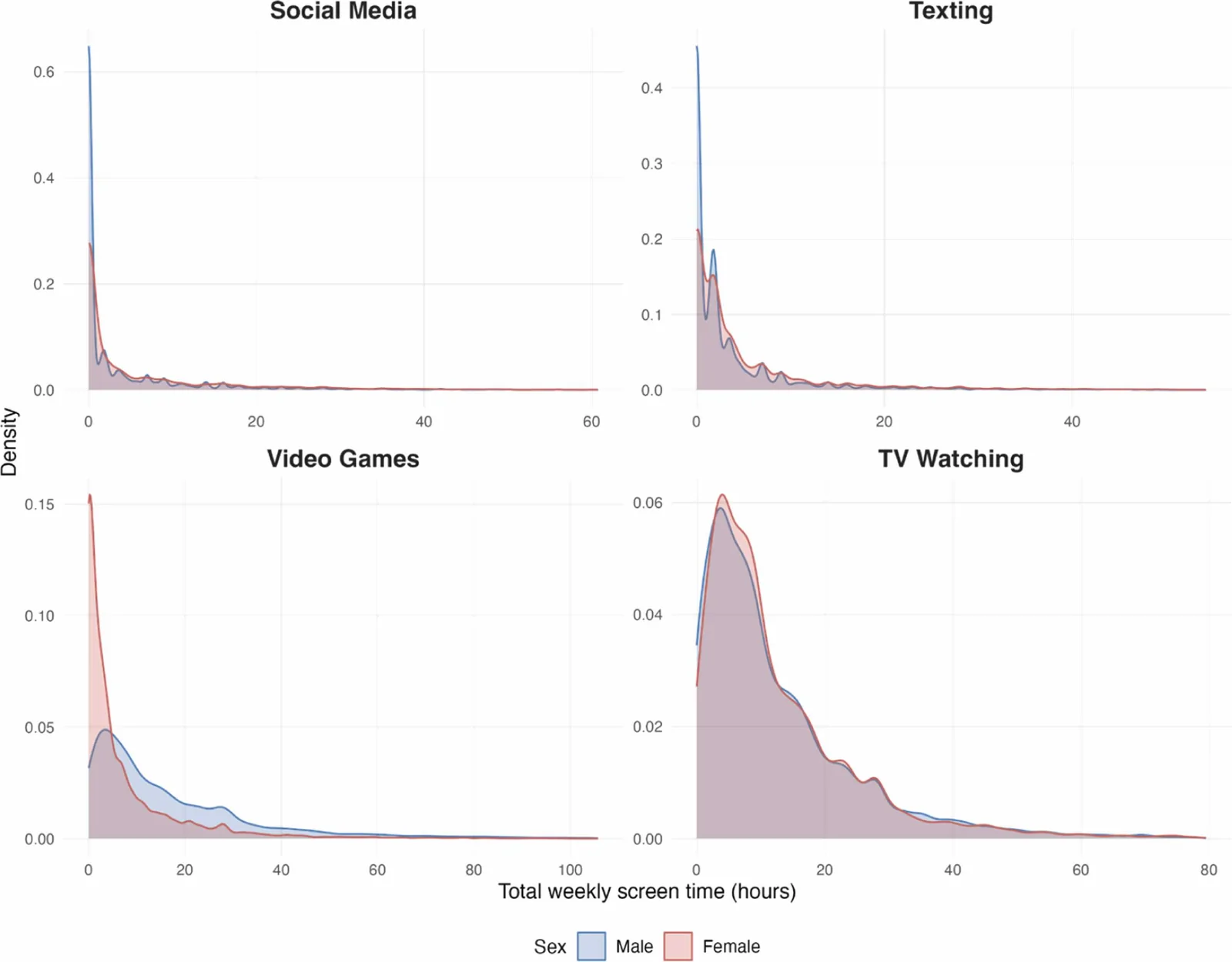
Distribution of weekly screen time activities by sex across four timepoints. Shaded areas represent where male and female distributions converge.

### 3.2 Nback neural signature associations with outcome variables

Higher accuracy on both 0-back (β = 0.39, CI= [0.38, 0.40], p < .0001) and 2-back (β = 0.25, CI= [0.24, 0.26], p < .0001) tasks was significantly associated with higher NS during WM.

On the other hand, engagement in screen time activities, including video gaming (β = −0.01, CI = [-0.03, −0.002], p = 0.02), texting (β = −0.026, CI = [-0.04, −0.01], p = 0.003), and television watching (β = −0.031, CI = [-0.047, −0.01], p = 0.0003), showed significant negative associations with NS scores and all the values survived FDR correction. Social media use was not significantly associated with NS (β=-0.011, CI = [-0.02, 0.0025], p = 0.11). Additionally, we confirmed that the association between brain activation and screen time is not driven by either the high-load (2-back) or the low-load (0-back) condition alone but is present in both conditions (see Supplementary Material S2). Further, the associations were largely specific to the neural systems engaged by the EN-back task: no associations were observed for the MID task, and for the SST, associations were limited to video gaming and social media use (incorrect-stop vs. correct-go contrast; see Supplementary Material S1).

### 3.3 Sex stratified analyses of NS associations with outcome measures

The distribution of screen time engagement varied by sex (Fig. 2). Among participants who engaged in each activity, video game playing showed the strongest male skew (60% male, 40% female), while social media use, texting and TV watching were relatively balanced (51.5% male, 48.5% female).

The regression analyses revealed distinct patterns of associations between the NS and screen time activities for each sex (Fig. 3). For females, none of the screen time activities showed significant associations with the NS (all β<-0.005, CI= [-0.03, 0.01], p ≥ .05). In contrast, males demonstrated significant negative associations across all screen time activities. Specifically, TV watching (β = −0.049, CI= [-0.07, −0.029], p < .001), texting (β = −0.041, CI= [-0.06, −0.02], p < .001), video game playing (β = −0.022, CI= [-0.04,-0.001], p = .028), and social media use (β = −0.022, CI=[-0.04, −0.002], p = .033,) were all significantly associated with the NS and all the values survived FDR correction.

**Fig. 3:**
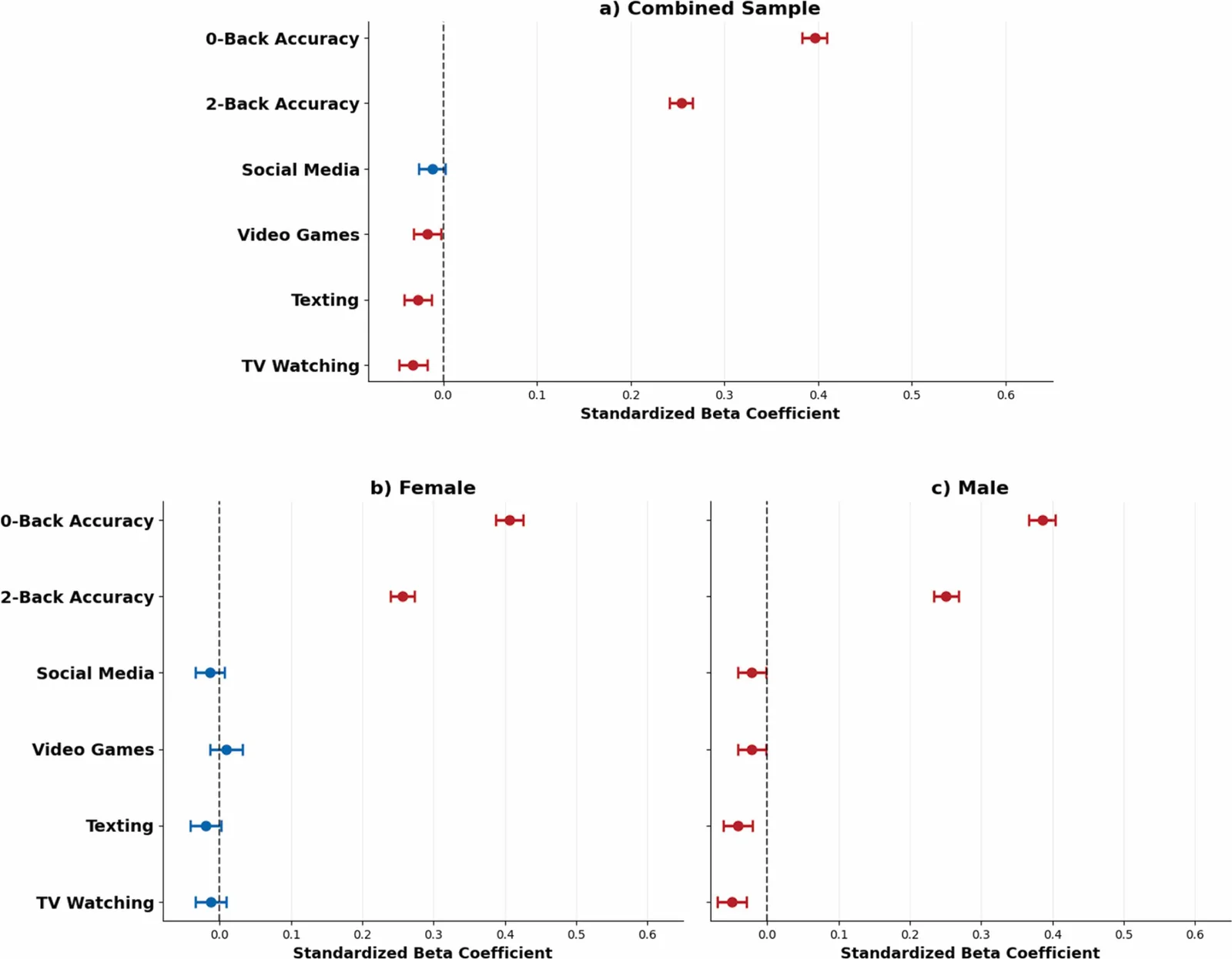
Associations between EN-Back NS scores and screen time activities and task performance. Points represent standardized beta coefficients and error bars indicate 95% confidence intervals from covariate-adjusted models. Points in red are statistically significant. Results are shown for (a) the combined sample, (b) females, and (c) males. The dashed vertical line indicates no association (β = 0).

### 3.4 Three-way interaction analyses among NS, screen time and mental health measures

#### 3.4.1 Externalizing behaviors

Three-way interaction analyses revealed that family conflict and physical activity nominally significantly moderated the relationship between the NS and SMA on externalizing behaviors. Specifically, family conflict moderated the NS × social media interaction (β = 0.01, CI = [0.0001, 0.02], p = .024, FDR-p > .05), NS × texting interaction (β = 0.016, CI = [0.002, 0.03], p = .01, FDR-p > .05) and physical activity moderated the NS x social media (β = 0.009, CI = [0.0002, 0.01], p = .043, FDR-p > .05). These three way interactions are plotted in Fig. 4. Additionally, significant associations were observed for the NS × family conflict and NS × sleep disturbance interactions (β >-0.23, CI = [-0.34, −0.02], p < .01, FDR-p < .05).

**Fig. 4:**
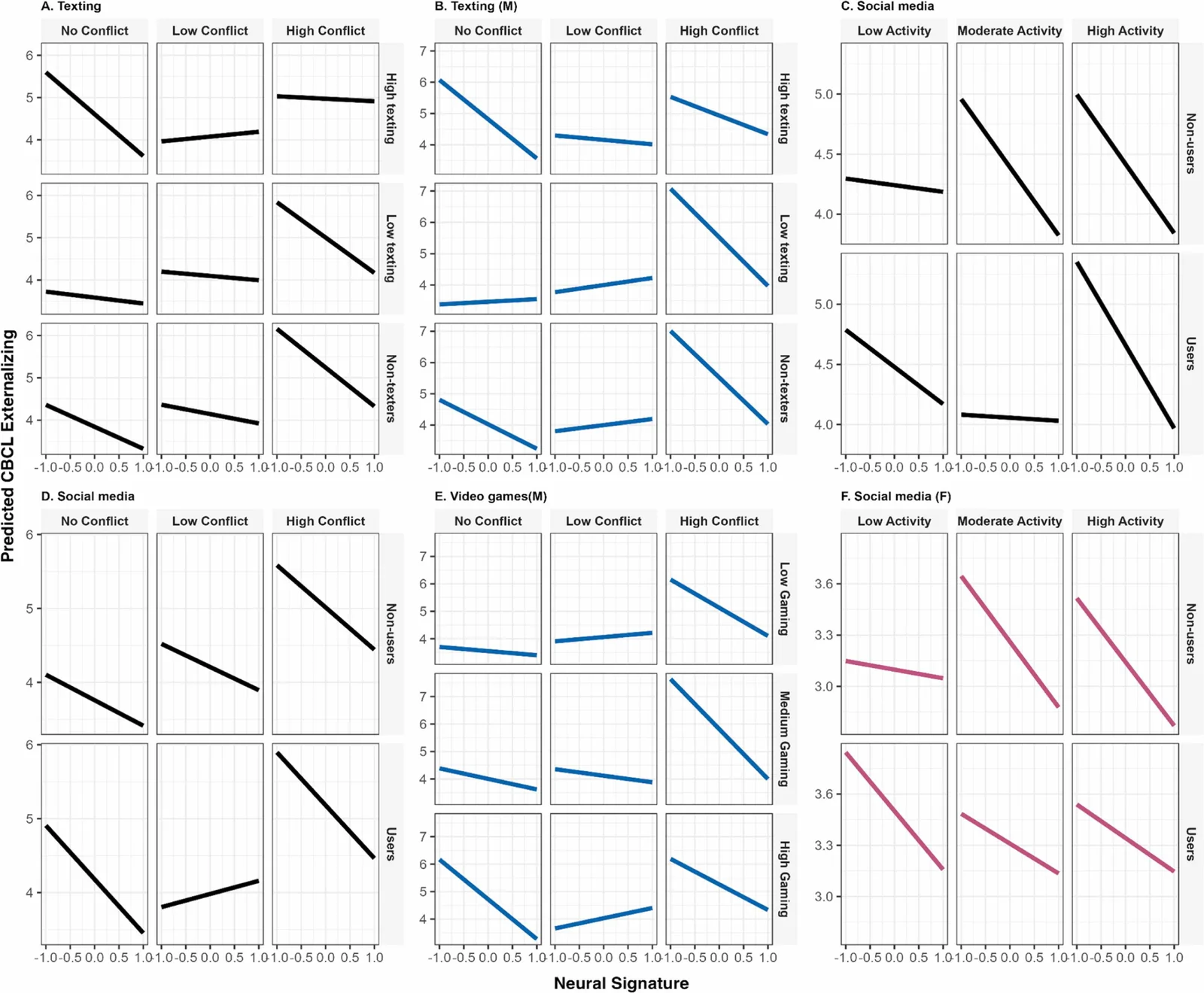
Three-way interactions among neural signatures (NS), screen media activities, and moderators (family conflict or physical activity) predicting CBCL Externalizing scores. Panels A–B and D–E depict interactions with family conflict (No/Low/High Conflict), while panels C and F depict interactions with physical activity (Low/Moderate/High Activity). Panel rows within each plot reflect tertiles of the corresponding screen activity (e.g., Low/Medium/High Gaming, High/Low texting, Users/Non-users). Black lines represent the combined male–female sample (Panels A, C, D); blue lines represent male-only analyses (Panels B, E); pink lines represent female-only analyses (Panel F).

Sex-stratified analyses revealed distinct patterns of moderation. Among males, family conflict significantly moderated the NS x video games interaction (β = 0.01, CI = [0.004, 0.02], p = .004, FDR-p < .05), NS × texting interaction (β = 0.04, CI = [0.01, 0.06], p = 0.001, FDR-p < .05) and physical activity moderated the NS x social media (β = 0.01, CI = [0.001, 0.02], p = .02, FDR-p > .05) in females. Furthermore, the NS × family conflict (β= −0.36, CI = [-0.57, −0.16], p = .0004, FDR-p < .05) and NS × sleep disturbance (β = −0.57, CI = [-0.98, −0.16], p = .0057, FDR-p < .05) interactions were significant in males.

#### 3.4.2 Internalizing behaviors

For internalizing behaviors, significant two-way interactions were observed between the NS and social media (β = 0.035, CI = [0.013, 0.06], p = .006, FDR-p < .05) and between the NS and texting (β = 0.043, CI= [0.01, 0.07], p = .0046, FDR-p < .05). Additionally, the NS × physical activity (β = −0.13, CI = [-0.23, −0.03], p = 0.009, FDR-p < .05) interaction was significant.

Sex-stratified analyses showed that among males, sleep disturbance nominally moderated the NS × videogames interaction (β = −0.01, CI = [-0.03, −0.001], p = .035, FDR-p > .05) (Fig. 5), and the two-way NS × social media interaction was nominally significant (β = 0.03, CI = [0.001, 0.07], p = .041, FDR-p > .05). Among females, nominally significant two-way interactions emerged for NS × TV watching (β = 0.03, CI = [0.002, 0.05], p = .03, FDR-p > .05).

**Fig. 5:**
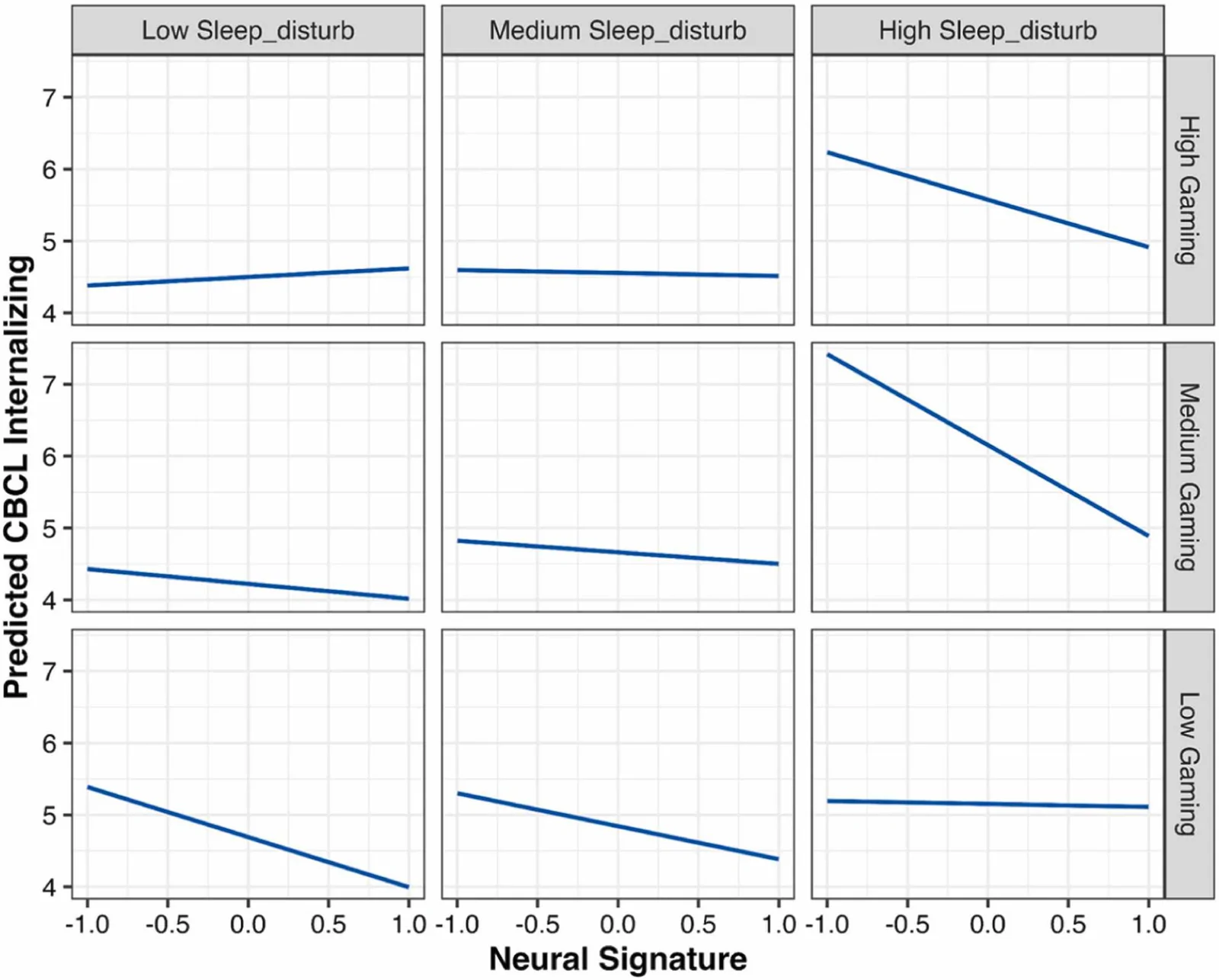
Multiple plots illustrating three-way interactions among NS, Video gaming, and sleep disturbance on internalizing stratified by sex. *Blue lines represent the male-only analyses.*

### 3.5 Impact of cognitive ability

In response to a reviewer’s comment, the above stated associations were confirmed to remain significant in the combined, sex-stratified and interaction analyses whether or not general cognitive ability (NIH Toolbox total composite score) is accounted for, indicating that they are specific to WM-related neural function rather than to general cognitive ability. NS was moderately correlated with general cognitive ability (r = 0.35; ~12% shared variance), indicating that most of the variance in NS is not accounted for by general cognitive ability.

## 4 Discussion

Using longitudinal data from the ABCD Study across four timepoints (ages 9–16), we examined associations among SMA, brain function and key moderating factors. We applied a NS approach (Baranger et al., 31, 2025) shown to improve reliability of neurobiological correlates of individual difference measures. Higher NS scores were associated with better cognitive performance whereas greater engagement in video gaming, texting, and TV watching was associated with lower NS scores, particularly among males. Family conflict, sleep disturbance, and physical activity moderated relationships between NS, screen time, and internalizing and externalizing symptoms, with sex-specific patterns.

The neurobehavioral findings of higher accuracy (Baranger et al., 31, 2025) on both the 0-back and 2-back conditions associated with higher NS suggest that better WM performance reflects more separable neural representations across task loads. This aligns with developmental fMRI evidence that successful EN-back performance depends on load-sensitive engagement of distributed frontoparietal control systems (Rosenberg et al., 2020, Satterthwaite et al., 2013). For example, EN-back task findings indicate that individual differences in WM ability are reflected in the magnitude and spatial distribution of frontoparietal activation, with stronger task-evoked responses predicting better out-of-scanner WM performance (Rosenberg et al., 2020). Recent work shows that the frontal cortex flexibly adapts its computational role for WM storage depending on specific task demands (Shao et al., 14, 2025), supporting the interpretation that higher NS scores reflect more efficient working-memory-related function. Also, WM development involves increasingly refined recruitment of cognitive circuitry (Satterthwaite et al., 2013).

In addition, we found that heavier exposure to video gaming, texting, and television watching is linked to lower NS reflecting reduced neural differentiation across WM load conditions. Although consistent with evidence linking higher SMA to less efficient cognitive control (Marciano et al., 2021), prior findings remain mixed: several studies report null or positive associations, particularly for gaming, which has been linked to small cognitive benefits and altered cortical pathways (Chaarani et al., 2022). Longitudinal work further suggests that higher television and video-game use predicts better WM in young adult males but not females (Soares et al., 2021). These findings suggest few possibilities: (1) NS captures neural efficiency beyond behavioral performance alone; (2) media content and context (e.g., competitive gaming vs. passive viewing vs. social communication) produce divergent trajectories; and (3) developmental timing influences outcomes. In addition, environmental factors such as family environment and sleep may further moderate neural associations. Notably, prior studies have not used multivariate NS methods in large population-based samples. By applying a NS approach across up to four timepoints from the ABCD Study, the present findings extend prior work by highlighting differences in the neural implementation of WM that may not be evident from behavior alone.

The interaction findings further indicate that family conflict significantly moderated associations between NS, texting and video gaming, and externalizing behaviors, particularly among males; these patterns were absent in females. Consistent with prior work, family conflict was associated with externalizing behaviors, amplifying associations between environmental exposures and behavioral dysregulation, (Lee et al., 2025) especially in males (Brieant and Simmons, 2025, Morelli et al., 2022). Under high family conflict, greater gaming was associated with elevated externalizing symptoms, aligning with evidence that excessive gaming in stressful contexts may exacerbate behavioral regulation (Prescott et al., 2018). Notably, this association was absent under low family conflict. In contrast, higher texting under high family conflict was associated with lower externalizing symptoms among youth with higher NS scores, suggesting a potential buffering role of digitally mediated social interaction. This aligns with findings that online communication and peer connectedness can provide social support and emotional regulation during family stress (Yau et al., 2021, Liu et al., 2019).

Beyond SMA-specific interactions, sleep disturbance and physical activity moderated associations between neural function and CBCL internalizing and externalizing symptoms. Physical activity strengthened the protective association between higher NS scores and lower internalizing symptoms, consistent with evidence that physical activity reduces internalizing problems in adolescence through neurobiological and psychosocial mechanisms (Yang and Sun, 2025). In contrast, sleep disturbance amplified the association between lower NS and higher externalizing symptoms, indicating that disrupted sleep is associated with greater vulnerability in youth with weaker WM-related neural engagement while those with stronger engagement show comparatively fewer symptoms. This pattern aligns with evidence linking sleep difficulties and both externalizing and internalizing problems in youth with mental illness (van Tetering et al., 2025). Given links between screen time and ADHD-related brain pathways (Shou et al., 2025), these findings suggest that regulatory factors like sleep and physical activity are associated with how WM-related neural function relates to CBCL internalizing and externalizing symptoms. Further, given the small effect sizes and the use of broad outcome measures (CBCL broadband scales and EN-back accuracy), these findings should be interpreted with caution. Broader claims regarding neurocognitive development and overall mental health will require validation through more fine-grained cognitive and psychiatric assessments.

Finally, interactions among NS and social media use and texting indicate that the association between digitally mediated communication and behavioral outcomes depend on WM-related neural function. This aligns with evidence that mental-health effects of social media are shaped by individual differences rather than uniform risk processes (Fassi et al., 2025, Myruski et al., 2024). Adolescents with stronger WM-related neural engagement may be more sensitive to socially evaluative content, strengthening associations with internalizing symptoms. Conversely, youth with weaker neural engagement may process these interactions less intensely, attenuating emotional impact. This interpretation is consistent with work showing that cognitive control, emotion regulation, and stress sensitivity moderate associations between digital media use and anxiety or depression (Shannon et al., 2022).

## 5 Limitations

Some limitations should be considered when interpreting these findings. SMA measures exhibited non-normal distributions with substantial zero inflation, which may have reduced sensitivity to detect associations across the full range of exposure. The weekday SMA data were not collected at the Year 6 timepoint, limiting our ability to fully characterize longitudinal patterns of screen use across all four timepoints. Although many observed associations were statistically significant, effect sizes were small. However, small effect sizes (Dick et al., 2021) are expected in large, population-based developmental cohorts such as the ABCD Study given the complex, distributed, and multilevel determinants of brain and behavioral outcomes and can nonetheless represent meaningful and reproducible effects at the population level, particularly when measurement reliability is high and findings are consistent across modalities and timepoints. Finally, even though the classifier was trained to differentiate the high-load WM (2-back condition) from the low-load (0-back condition), this should not be confused with the 2vs0 back contrast derived from the standard GLM which is generally used by researchers, and which may yield different outcomes.

## 6 Conclusion

Overall, we demonstrate that SMA is associated with WM related brain function, with moderation by family environment, sleep, and physical activity. These findings highlight the role of modifiable contextual factors to developmental outcomes. Future work using upcoming ABCD data releases, including additional follow-up timepoints, will help clarify developmental trajectories and potential sensitive periods. Although brain-based and mental health outcomes may not be directly modifiable, the present findings identify actionable targets such as reducing family conflict, improving sleep quality, and promoting physical activity that can inform prevention strategies and policy efforts to support adolescent neurocognitive and mental health development.

## CRediT authorship contribution statement

**Sahithi Arekatla:** Writing – review & editing, Writing – original draft, Visualization, Validation, Methodology, Formal analysis, Data curation. **Anthony Barrows:** Writing – review & editing, Methodology, Formal analysis. **Leigh-Anne Cioffredi:** Writing – review & editing, Writing – original draft, Conceptualization. **Matthew D. Albaugh:** Writing – review & editing, Validation, Conceptualization. **Nicholas Allgaier:** Writing – review & editing, Software, Methodology. **Mckay Allred:** Writing – review & editing, Validation. **David Baranger:** Writing – review & editing, Methodology. **Alexandra Potter:** Writing – review & editing, Funding acquisition. **Hugh P. Garavan:** Writing – review & editing, Resources, Methodology, Funding acquisition. **Bader Chaarani:** Writing – review & editing, Writing – original draft, Visualization, Validation, Supervision, Project administration, Methodology, Funding acquisition, Formal analysis, Data curation.

## Declaration of Competing Interest

The authors declare that they have no known competing financial interests or personal relationships that could have appeared to influence the work reported in this paper.
