# Supplementary material for: Longitudinal associations between screen time activities and working memory neural signatures in adolescents: Sex differences and contextual moderation
Source: Dev Cogn Neurosci. 2026 Oct 6;82:101836. doi: 10.1016/j.dcn.2026.101836 (PMC13678101; doi:10.1016/j.dcn.2026.101836)
Supplement: Supplementary file 1 [file mmc1.docx]

**Supplementary Material**

**Longitudinal Associations Between Screen Time Activities and Working Memory Neural Signatures in Adolescents: Sex Differences and Contextual Moderation**

Sahithi Arekatla, Anthony Barrows, Leigh-Anne Cioffredi, Matthew Albaugh, Nicholas Allgaier, McKay Allred, David Baranger, Alexandra Potter, Hugh Garavan, Bader Chaarani

S1. Specificity Analyses Using Stop-Signal Task and Monetary Incentive Delay Task Neural Signatures

Fig. S1. Associations Between SST Neural Signature Scores and Screen Time Activities and Task Performance

**S2. Univariate ROI Analyses of 2-back and 0-back Activation and Screen Time**

**Table 1.** Number of significant ROI-level associations between each screen media activity and brain activation in the 2-back and 0-back conditions.

This supplementary material has been provided by the authors to give readers additional information about their work.

# **S1.** Associations Between Neural Signatures and Screen Time in SST and MID Tasks

To check whether our findings are specific to working-memory-related brain function, rather than a general task-related brain activation, we created NS for two other ABCD fMRI tasks that measure different cognitive processes: the Stop-Signal Task (SST), which measures response inhibition, and the Monetary Incentive Delay (MID) task, which measures reward anticipation and feedback processing. We tested whether these task-based measures were linked to the same four screen time activities, using the same covariates as our main models, including general cognitive ability.

For the SST, television watching and texting showed no association with neural signature scores derived from correct-stop vs. correct-go or incorrect-stop vs. correct-go contrasts (all p > .13). However, video gaming and social media use were associated with neural signature scores from the incorrect-stop vs. correct-go contrast in the full sample with Videogaming (β = -0.01, CI = [-0.028, -0.0023], p = .02, FDR-p<.05), social media (β = -0.02, CI = [-0.03,- 0.008], p = .001, FDR-p<.05) and in males with Videogaming (β = -0.02, CI = [-0.043, -0.006], p = .008, FDR-p<.05) and social media (β = -0.02, CI = [-0.043, -0.007], p = .006, FDR-p<.05) with no significant associations in females. For the MID task, neither neural signatures for the reward anticipation vs. neutral anticipation contrast, nor reward positive feedback vs. neutral positive feedback contrasts were linked to any measure of screen time activity (all p > .16).

**Fig. S1.** Associations between Stop-Signal Task (SST) neural signature (NS) scores and screen time activities and task performance. Points represent standardized beta coefficients, and error bars indicate 95% confidence intervals from covariate-adjusted models. Points in red are statistically significant. Results are shown for (A) the combined sample, (B) females, and (C) males. The dashed vertical line indicates no association (β = 0).

**S2. Associations Between specific condition ROI Activation and Screen Time in the EN-back Task**

To examine whether the associations between screen media activity (SMA) and the neural signature (NS) were driven by one task condition, we tested the association between SMA and ROI-level beta weights from the standard EN-back contrasts used in prior ABCD studies, separately for 2-back vs. fixation and 0-back vs. fixation. Analyses focused on a subset of 12 bilateral ROIs that were most active during the task and contributed most strongly to the NS classifier's predictions (Figure 1 in the main text). These included frontomarginal, subcentral, cingulate, middle frontal, temporal, parietal, postcentral, precuneus, calcarine, and hippocampal regions.

After FDR correction (p < .05), a comparable number of ROI-level associations with screen time were significant in the 2-back (37 of 96 tests) and 0-back (39 of 96 tests) conditions. This pattern was consistent across SMA types (Table 1). For example, texting was associated with activation in 14 ROIs in the 2-back condition and 16 ROIs in the 0-back condition, and video gaming with 8 ROIs in the 2-back condition and 6 in the 0-back condition. These results indicate that the association between brain activation and screen time is not driven by either the high-load (2-back) or the low-load (0-back) condition alone but is present in both conditions.

| SMA / Number of significant ROIs |  |  | 2-back | 0-back |
| --- | --- | --- | --- | --- |
| TV |  |  | 8 | 8 |
| Texting |  |  | 14 | 16 |
| Video games |  |  | 8 | 6 |
| Social media |  |  | 7 | 9 |

**Table 1.** Number of significant ROI-level associations (FDR-corrected at *p*<.05) between each screen media activity and brain activation in the 2-back and 0-back conditions.
